# A Synergistic Approach to Atmospheric Water Scavenging

**DOI:** 10.1021/acsami.2c18920

**Published:** 2023-01-30

**Authors:** Shichao Jiao, Joseph J. McCarthy

**Affiliations:** Department of Chemical and Petroleum Engineering, University of Pittsburgh, Pittsburgh, Pennsylvania15261, United States

**Keywords:** water uptake, composite, capillary condensation, Kelvin equation, relative humidity, self-assembly

## Abstract

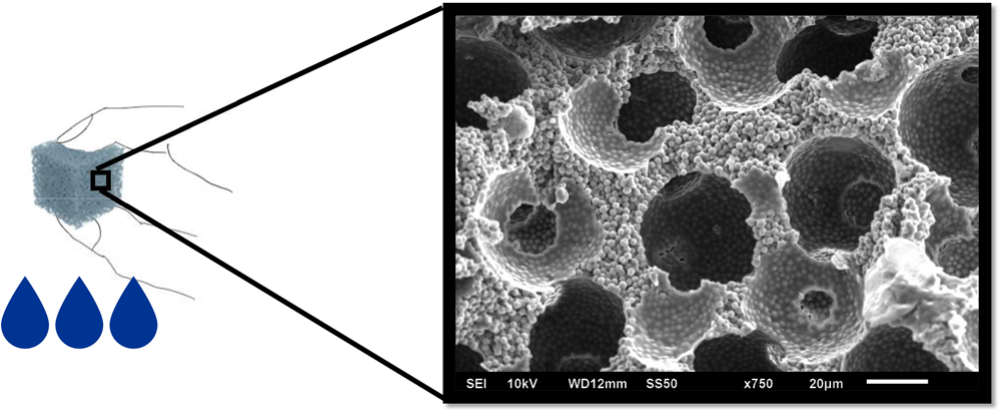

An abundant supply of fresh water is one of the leading challenges of the 21st century (UNESCO. The United Nations World Water Development Report 2018: Nature-Based Solutions for Water*;*UNESCO: Paris, France, 2018; p 154). Here we describe a new approach to scavenge atmospheric water that employs a hierarchically ordered porous material with embedded particles (LashM. H.; JordanJ. C.; BlevinsL. C.; FedorchakM. V.; LittleS. R.; McCarthyJ. J.Non-Brownian Particle-Based Materials with Microscale and Nanoscale Hierarchy. Ang. Chem. Int. Ed.201554, 5854−585810.1002/anie.20150027325892480). This composite uses structure to amplify native material performance to realize synergy between the capture and storage and to ultimately qualitatively change the adsorption behavior of the hydrogel (from unfavorable to favorable). In this way we can capture moisture at significantly lower relative humidities than would otherwise be feasible with the native materials. Not only does this approach pose the potential for a cheap and low-energy source of clean water but it could also be modified for application across a variety of condensable vapor reclamations.

## Introduction

For decades researchers from across the globe have examined a variety of techniques for generating clean water^3^ from desalination^4^ to disinfection/decontamination.^5,6^ More recently, a growing trend aims at harnessing the abundant supply of fresh water that is available within the atmosphere.^7^ To date, such water scavenging approaches have largely leveraged daily heating/cooling cycles to overcome the energetic cost of condensation.^8−10^ Some mimic the behavior of the desert beetle^11^ to capture early morning fog using, for example, synthetic netting^12^ or other biomimetic materials.^13^ Another technique exploits solar energy more directly in order to enhance the release of water captured within a metal–organic framework (MOF)-based sorbent material.^14,15^ The approach espoused here, inspired by granular flows,^16,17^ employs a novel composite material capable of passive capture via a capillary condensation process with subsequent low-energy reclamation of the water through simple finger pressure. Despite the complex microstructure of the composite and nanometer-sized length scales of the resultant contact spots,^18^ a continuum-based thermodynamic analysis accurately describes the observed results.

Even for particles that are hundreds of microns in diameter, surface asperities can cause aging of the material,^17^ which results in an increase in the static angle of repose of a granular bed. This phenomena was quantitatively described by Bocquet et al. using the Kelvin equation^19^ by attributing the angle increase to cohesion between the particles due to the capillary condensation of liquid bridges at the points of asperity contact. This tendency of particle imperfections to yield an order of magnitude decrease in the effective radius of curvature at contact spots is not only a boon to the longevity of sand castles^20^ but also forms the basis of the “capture” portion of our synergistic water scavenging approach.

It has long been recognized that nanometer-scale channels/curvature can nucleate capillary condensation,^21,22^ and it was recently shown that this phenomenon is quantitatively described by the Kelvin equation at scales even smaller than a nanometer.^18^ Nevertheless, exploiting this relationship for water scavenging purposes is hampered by several factors, including the cost of nanoscale fabrication, the storage capacity of the nanostructured devices, and the ultimate recovery of liquid water that collects at the contact spots. In contrast, hydrogels have been recognized for decades as an outstanding storage medium for large quantities of (liquid) water,^23^ allow water recovery from simple compression/squeezing,^24^ and have even been shown to absorb a modest amount of moisture directly from vapor streams.^25^

## Results and Discussion

**Figure 1:**
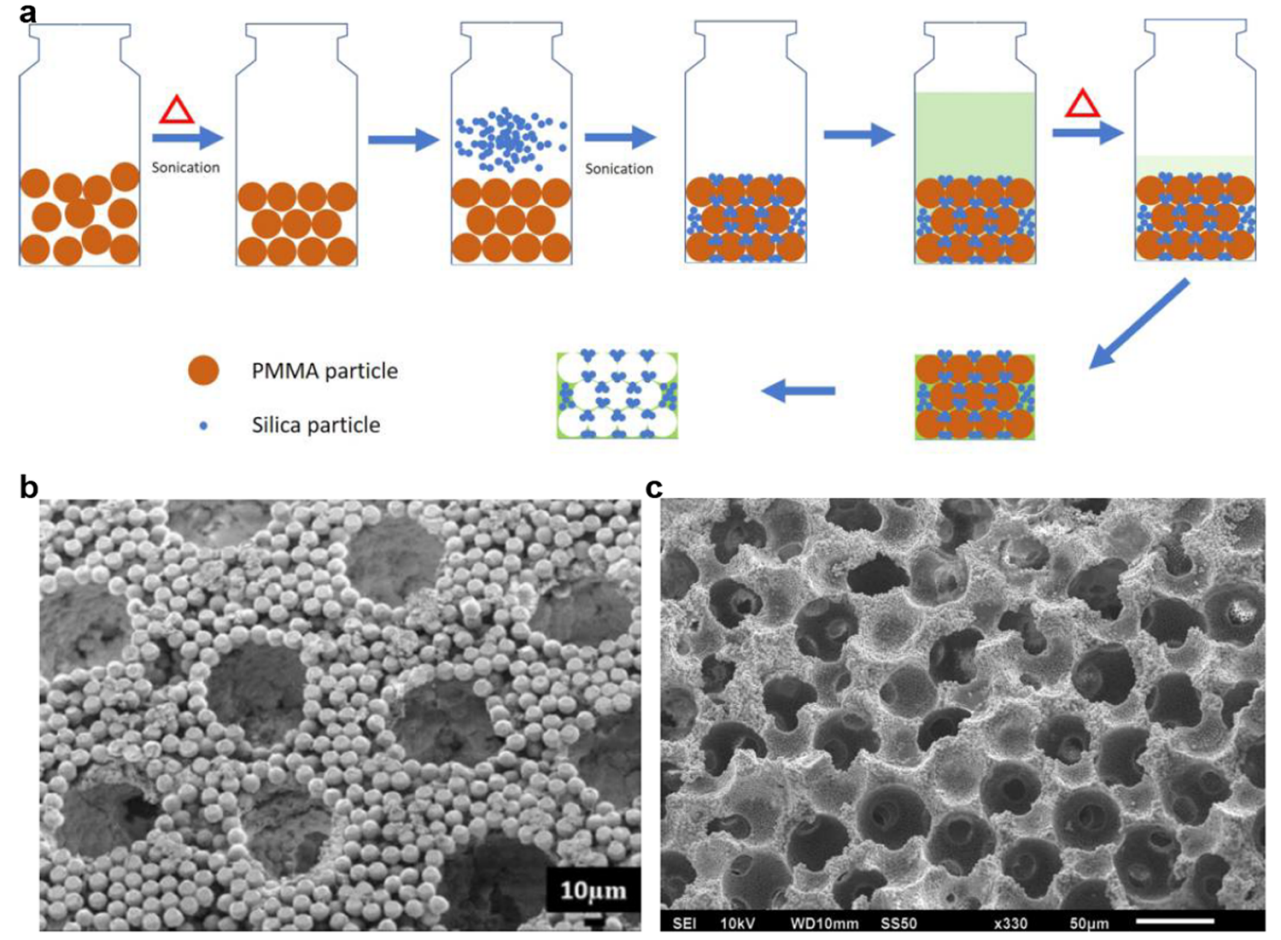
(a) Schematic of the synthesis of the composite. Sonication is used to form an ordered array of larger particles. Slight heating fuses the contact spots between large particles (ensuring future continuous void space). Small, hydrophilic particles then fill the interstices between the large particles. A hydrogel infuses the remaining voidage. When the large particles are selectively removed, a hierarchically ordered porous composite remains. (b) SEM picture of the binary hierarchically ordered particle matrix (when no hydrogel is used). (c) SEM picture of hierarchically ordered particle matrix with hydrogel backbone (i.e., the composite).

The synergistic water-scavenging approach espoused here is comprised of materials that allow alternatively “capture” and “storage/release” of moisture. As such, the composite examined here uses a particle-based structure to create locations for capillary condensation, directly stores the water from the condensation spot in a continuous hydrogel, and allows water to be recovered by simple methods such as hand squeezing. While the details are outlined in the Methods section included in the Supporting Information (and illustrated in Figure 1a), based on the work of Lash et al.,^2^ we can create a hierarchically ordered porous matrix that has a continuously connected pore structure, a cross-linked poly(hydroxyethyl methacrylate) (pHEMA) hydrogel backbone, and an ordered array of densely packed particles at the boundary of each pore wall (see Figure 1b,c). The idea behind our study is to examine the interplay between confinement-induced condensation and hydrogel swelling. As shown below, this cooperative behavior—induced through structural design—qualitatively changes the character and efficacy of water vapor absorption within the composite material and can form the basis of a new class of condensable vapor scavengers.

**Figure 2:**
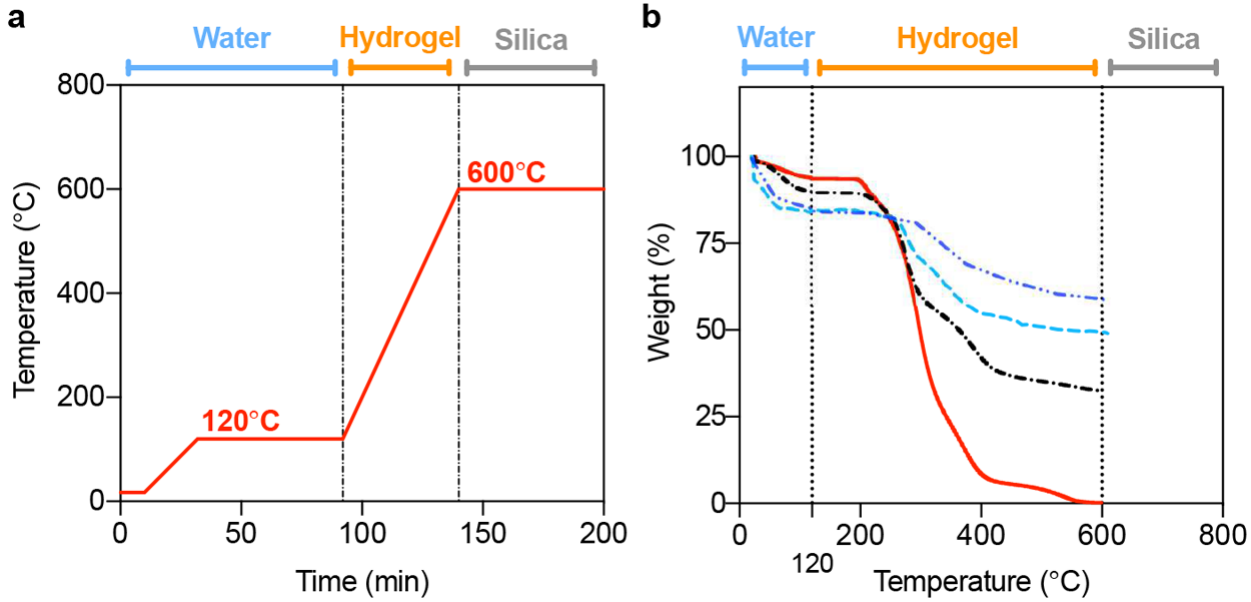
(a) The temperature ramping protocol used for TGA of the composite samples. (b) A series of TGA results for composites with different monomer concentrations and the pure hydrogel. The moisture content decreases and hydrogel content increases with increasing monomer concentration (ranging from 20% to 40%). The (red) solid line represents the thermal degradation curve of the pure hydrogel. The (blue) dash-dotted line, (blue) dashed line, and dash-dotted line represent the thermal degradation curves of the composites containing 20%, 25%, and 40% monomer concentration hydrogel, respectively. Based on these results, the composite containing a 25% monomer concentration hydrogel is used in the remainder of the study as a compromise between water absorption and structural integrity.

**Figure 3:**
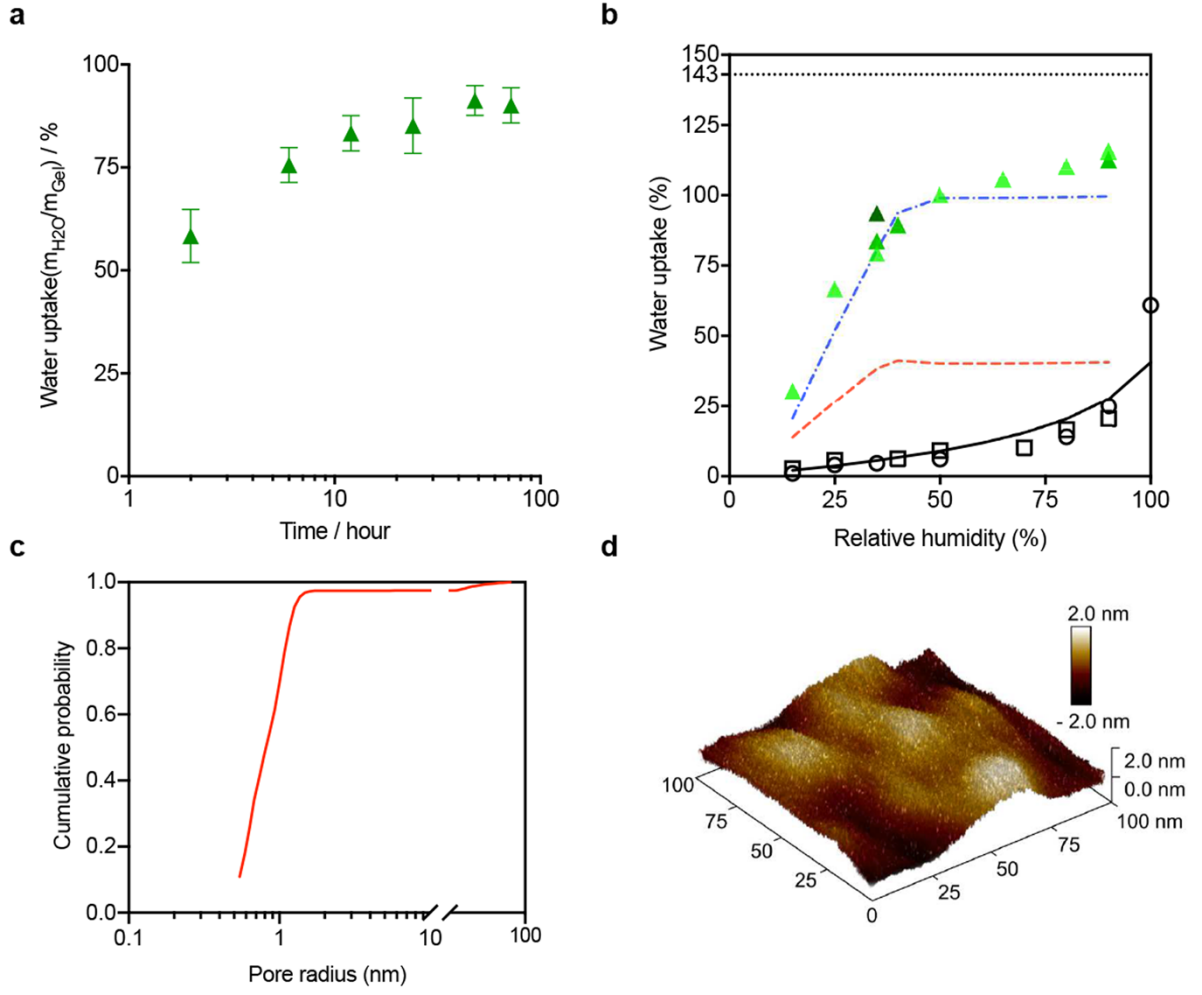
(a) Water uptake of the composite when exposed to a 35% RH environment over a period of many hours. We define water uptake as the ratio of absorbed water mass to hydrogel mass, expressed as a percentage. (b) Water retention isotherms for the composite (multiple results are presented) and pure porous pHEMA hydrogel and their corresponding theoretical curves when samples are exposed to vapors of varying RH for 48 h. Note that an immersed sample of pure hydrogel absorbs 143% of its own mass (shown as a dotted line). Open circles and squares represent a low and high porosity hydrogel sample, while (green) triangles represent composite results (with two darker shades corresponding to additional composite trials). The solid line represents the thermodynamic predictions of hydrogel-based vapor absorption, while the (red) dashed and (blue) dash-dotted line show the results from the composite theory and composite theory with pore filling, respectively. (c) BET surface area analysis for a composite sample, showing the cumulative distribution function (CDF) of pore sizes. (d) An AFM image of a 100 nm^2^ surface of a representative silica particle. Note that surface asperities include imperfections in the range of 1 nm in size.

In order to test the water-adsorptive capacity of our composite, we use a humidity-controlled glovebox. The material to be tested is placed in the box under different relative humidity (RH) environments, ranging from 15% to 90%. For each measurement, the sample was allowed to approach equilibrium over the course of a 2 day exposure. Figure 3a shows that, for a relative humidity of 35%, a 48 h exposure is sufficient to realize the asymptotic adsorption within the material. In addition to testing a variety of realizations of our composite material, as a control, we also tested several samples of porous pHEMA gel (see Methods section for the fabrication technique of both a high and low surface area porous gel; note that, in each of these samples, we have omitted the small silica particles). The mass of all tested samples is measured both pre- and postexposure (with samples sealed in an airtight bag for transport between the humidity chamber and scale). The samples were further evaluated using a thermogravimetric analysis method (TGA) in order to ascertain the absolute dry weight and composition of gel and silica particles within each sample (see Figure 2 and the Methods section for the TGA protocol). The absorptive performance is quantified based on the mass of water absorbed relative to both the mass of the total amount of hydrogel in that composite (in order to highlight the impact of structure on absorption).

In Figure 3b it can be seen that, under typical atmospheric conditions, the composite can recover from an ambient gas source nearly 80% of the water that would have been available from a liquid source (143% of the hydrogel weight). In contrast, the porous pHEMA hydrogel (as the control) can only achieve less than half of the maximum absorption under the same humidity conditions. More significantly, for the composite there is a sharp increase of water uptake observed near a relative humidity of between 25 and 30%, while the absorption of the control is far more gradual; thus, the control achieves an extremely low uptake at relative humidities below 50%.

According to Flory–Huggins theory,^26,27^ the equilibrium swelling of a cross-linked polymer network can be represented by
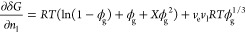
where *δG* is the Gibbs free energy change, *R* is the gas constant, *T* is the temperature, ϕ_g_ is the volume fraction of the gel in the mixture, χ is the Flory–Huggins parameter, *n*_l_ is the moles of the solvent, *v*_l_ is the molar volume of the solvent, and *v*_e_ is the moles of chains per volume.

In the case when water vapor is the source of the swelling solvent and is therefore in equilibrium with the swollen hydrogel, an additional term is required leading to a complete thermodynamic relationship for water absorption as shown below.
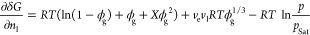


For our control samples, we can estimate the water volume fraction (hence the water uptake) at equilibrium by setting the free energy change to zero. Using a nonlinear curve fit for both the Flory–Huggins parameter χ as well as the chain density *v*_e_, using the experimental data for the porous pure pHEMA hydrogel experiment data (see Figure 3b, black line), we obtain the parameters of χ as 1.05 and *v*_e_ as 1.25 × 10^–4^ mol/ml. In order to use this model for our composite material, we must recognize that the nucleation sites for capillary condensation that are inherent in the structure of our material will alter the vapor-equilibrium term of this equation. That is, we must use the Kelvin equation^27^ near nucleation sites so that we modify the effective location saturation pressure from that of the “flat” value (*p*_Sat_) to that of the curved value (*p*_Sat_^C^).
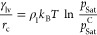


Here, *r*_c_ represents the radius of the curvature near the contact spots. Using this equation, we note the critical curvature values *r*_c_ that would result in an effective local relative humidity of 100% (i.e., for nonconfined relative humidities below 50%, we require condensation spots in our composite that have a radius of curvature less than 1.5 nm). Using a Brunauer–Emmett–Teller (BET) measurement of our composite, we find the pore size distribution of the composite. Figure 3c confirms that most of the pores in the composite have a diameter less than 1.5 nm. Moreover, an atomic force microscope (AFM) image (Figure 3d) of the surface of our silica particle inclusions confirms the asperity scale to coincide with this size. By assuming that these pores are uniformly distributed throughout the composite, we can apply our simple thermodynamic approach using a nonconfined relative humidity for the fractions of the composite whose pore curvatures *r* are larger than the critical value *r*_c_ but instead assume saturated conditions for the fractions where *r* < *r*_c_. The (red) dashed curve in Figure 3b shows the qualitative change in absorption behavior under these conditions. Despite this modification to our theoretical approach, there remains a quantitative difference between the experimental measurements (triangles) and this modified theory (Figure 3b). This discrepancy stems from the lack of consideration of free moisture filling the pore spaces near the condensation spots. That is, the modified theory allows for hydrogel equilibration with free moisture, but the model does not account for the remaining free moisture. In order to estimate the amount of water trapped by filling the (correctly sized) pore spaces with free moisture we again turn to the measurement of the cumulative pore size distribution (Figure 3c). By using the fraction of the total open pore volume that has curvature sufficient to induce free moisture condensation, along with the experimental measurement of the swelling ratio (i.e., the product of the gel density and the maximum water uptake, which yields 1.65 g of water per milliliter of gel), we are able to calculate the free moisture trapped within the pore spaces at each relative humidity (shown as the (blue) dash-dotted line in Figure 3b). Note that, by accounting for both effects of the local confinement, we obtain a modified model that matches experimental measurements quite closely.

**Figure 4:**
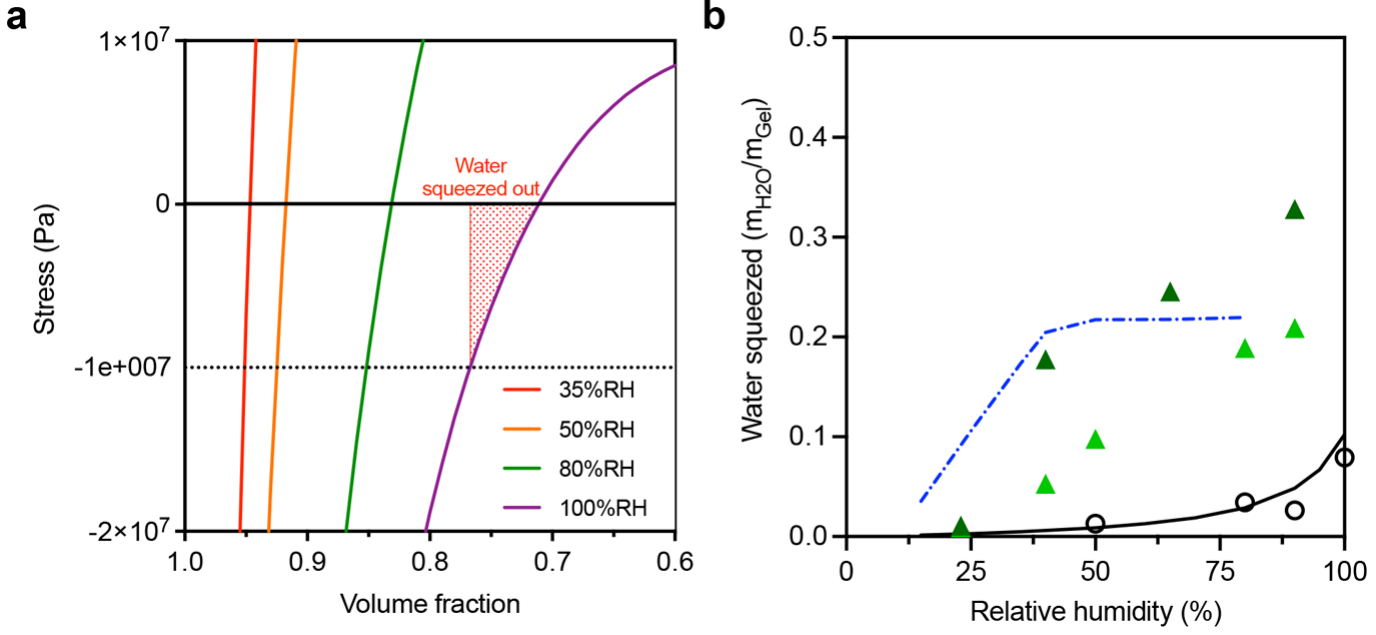
(a) Theoretical relationship between applied pressure and hydrogel volume fraction for a series of RH values (humidity increases for curves from left to right) based on the thermodynamic analysis included in the text. Note that water uptake (in volume fraction units) may be obtained from the 0 Pa stress crossing, while water expulsion upon squeezing is obtained from negative stress values (here taken to be approximately −7 × 10^7^ Pa). (b) Mass fraction of water squeezed out of both composite and hydrogel samples, under different RH environments. Here we express results as a ratio of the mass of water expulsion to the hydrogel mass. Open circles represent a pure porous hydrogel sample, while (green) triangles represent two realizations of composite material. As in Figure 3, the solid line corresponds to the thermodynamic analysis, while the (blue) dash-dotted line represents the modified version applicable to the composite.

To confirm that the increased water uptake is not simply attributable to the excess pore-filling outlined in Figure 3b, we conducted a sequence of “component” tests, as follows. We first tested the bare pHEMA hydrogel under 93% RH. Under these conditions, the hydrogel absorbed 101.9 mg of water, representing 13.21% of its dry mass (which was 771.4 mg). Similarly, when we deposited a layer of bare silica particles onto a silicon wafer, the system absorbed 3.5 mg of water, representing 219% of the particles’ (dry) mass (which was 1.6 mg). We then combine these two components by forming a pHEMA hydrogel film on top of particles that were deposited on the silicon wafer and peel off the film to expose a composite surface to the moist air. A naive superposition of the component absorptions would suggest that this composite would yield 41.4 mg of water (based on the absorption expected from the 1.6 mg of particles embedded in 286.8 mg of pHEMA). Interestingly, we instead observe that this composite film absorbs 68.2 mg, so that the synergistic effect of combining the moisture capture and storage yields a 65% increase in absorption efficacy.

As the final factor in understanding the behavior of our composite, we must investigate the response of the system to externally applied stress/pressure. Here, we introduce a stress term to our modified theoretical treatment (σ).^26,27^ The inclusion of an external stress changes the chemical potential of the solvent so that the relationship between the applied stress and the volume fraction of the gel at equilibrium is now expressed as
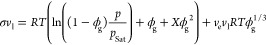


Figure 4a shows the relationship between the applied stress and the hydrogel volume fraction for a series of relative humidity values. By comparing the change in the volume fraction of the hydrogel between no externally applied stress (0 Pa) and an estimate of hand grip pressure (10^7^ Pa),^28^ we can suggest the amount of water that can be recovered by squeezing the sample. As can be seen from this analysis, with an increase of RH, the amount of water that can be recovered (i.e., the shaded area) increases. Comparing the experimental values to those predicted from this analysis shows substantial agreement from the samples of pure, porous hydrogel (open circles and the solid line, respectively). In order to analyze the composite, we apply the modified theory with both unstressed and hand-grip pressure to obtain the (blue) dash-dotted line. Note that, due to the very high Laplace pressures within most of the highly confined pore spaces, we assume that water is expelled almost exclusively from the hydrogel itself rather than from the pores (with the exception of the pores above 5 nm where the hand-grip pressure exceeds the Laplace pressure).

## Conclusion

Our composite shows great potential for scavenging of ambient water vapor and other condensable vapors in an economical, environmentally friendly, and remarkably simple way. The absorption process is completely passive in that it does not require external energy, special equipment, or any particular environmental conditions in order to function. Compared to most existing approaches using current absorbents, it is the structure of our composite that leads to a qualitative change in the absorption behavior of the ultimate material. This new structure greatly increases the efficiency of absorption when compared to the native material. Thus, the same central idea, that structure can be used to amplify native material performance, may be applicable to a variety of adsorbent materials. Even without optimization of the composite or fabrication, we note that up to 5% of the composite’s mass is easily recoverable at humidities below 50% from materials that are extremely abundant and inexpensive.

## Data Availability

All data is available in the manuscript or the Supporting Information.

## Supporting Information Available

Experimental methods and characterizations (ZIP)
